# Prediction of risk stratification in gastrointestinal stromal tumors using electron density combined with clinical and imaging features from spectral CT

**DOI:** 10.1186/s12880-026-02672-x

**Published:** 2026-08-07

**Authors:** Li-Ping Zhang, Xiu-Yu Wang, Xiao-Min Liu, Liang-Cai Li, Fang-Yun Li, Yong Quan, Bing-Hang Tang

**Affiliations:** 1 Department of Radiology, Zhongshan City People’s Hospital, No. 2 Sunwendong Street, Zhongshan, 528403 China; 2 Clinical and Technical Support, Philips HealthCare, Guangzhou, 510220 China

**Keywords:** Gastrointestinal stromal tumors, Tomography, X-ray computed, Risk classification, Electron density

## Abstract

**Background:**

Dual-layer spectral detector computed tomography (DLCT) generates multiple quantitative parameters, such as CT values on virtual non-contrast (VNC) images, iodine concentration (IC), electron density (ED), effective atomic number (Zeff), and CT values on virtual mono-energetic images (VMI), offering new insights for the diagnosis and evaluation of gastrointestinal stromal tumors (GISTs). This study aimed to develop and validate a multimodal predictive model that integrates dual-phase spectral CT parameters with key clinical-radiological features for preoperative risk stratification in GISTs.

**Methods:**

This retrospective study included 130 patients with pathologically confirmed GISTs, comprising 83 low-risk and 47 high-risk cases. Clinical data, traditional CT features and spectral CT quantitative parameters of electron density (ED), virtual non-contrast (VNC) images and spectral curve slope (λ) in both arterial phase (AP) and venous phase (VP) were collected and compared between the two groups. Least absolute shrinkage and selection operator (Lasso) was used to screen predictive indicators to build a logistic model. A nomogram was constructed based on the selected features, and model performance was evaluated using the receiver operating characteristic (ROC) curve and decision curve analysis (DCA).

**Result:**

The mean age was significantly lower in the low-risk group (56.6 ± 10.8 years) than in the high-risk group (61.3 ± 12.5 years) (P < 0.05). Tumor maximum diameter, necrosis occurrence rates, enlarged vessels feeding or draining the mass (EVFDM) occurrence rates were significantly higher in the high-risk group than the low-risk group (6.7 cm [4.2-8.6] vs. 1.6 cm [1.1–3.1], 72.3% vs. 12.0%, 63.8% vs. 10.8%, respectively; all P < 0.05). ED, VNC in arterial phase also showed significant differences between low-risk and high-risk groups (ED: 104.3 ± 1.0 vs. 103.5 ± 0.53; VNC: 39.7 ± 8.4 HU vs. 36.3 ± 7.1 HU; all P < 0.05). Based on Lasso regression, five features (tumor size, location, necrosis, EVFDM, and AP-ED) were used to construct the integrated model. The model demonstrated strong diagnostic performance, with an AUC of 0.936, sensitivity of 88.0%, specificity of 91.5%, and accuracy of 89.2%.

**Conclusion:**

A multimodal predictive model combining spectral CT–derived electron density with selected clinical and radiological features enables accurate preoperative risk stratification of GISTs, and may assist in guiding individualized management strategies.

## Background

Gastrointestinal stromal tumors (GISTs) originate from the interstitial cells of Cajal or their precursor cells in the gastrointestinal mesenchymal tissue, with an annual incidence of approximately 1–2 cases per 100,000 population [1]. GISTs can occur anywhere in the gastrointestinal tract, most commonly in the stomach (~ 60–70%), followed by the small intestine (~ 20–30%), infrequently in the colon, rectum, or esophagus [2]. The modified National Institutes of Health (NIH) criteria [3, 4] classify GISTs into very low-risk, low-risk, intermediate-risk, and high-risk groups, which are widely used for prognostic assessment. The current classification criteria for GISTs primarily rely on postoperative pathology, however, not all patients are suitable for this invasive procedure. Highlighting the urgent need for a clearer and more comprehensive preoperative evaluation standard. Preoperative risk assessment is crucial for determining treatment options and predicting prognosis. Some researchers have utilized imaging features to develop prediction models for GIST malignant potential [5–7].

Current preoperative evaluation of GISTs primarily relies on endoscopic ultrasound (EUS), conventional CT, and MRI. While EUS allows direct visualization of mucosal lesions and guided biopsy, its accuracy in assessing deep-seated or extraluminal tumors remains suboptimal (sensitivity 75–82%) [8]. Conventional CT provides excellent anatomical delineation but lacks functional parameters for characterizing tumor heterogeneity. MRI demonstrates superior soft tissue contrast yet suffers from prolonged acquisition time and motion artifacts in upper abdominal lesions. Although 18 F-FDG PET/CT shows potential in metabolic evaluation, its high radiation dose (effective dose ~ 30mSv) [9] and limited availability restrict routine clinical application. These limitations underscore the need for advanced imaging biomarkers that can noninvasively quantify tumor biological characteristics.

Dual-layer spectral detector computed tomography (DLCT) as a novel imaging technology, provides multiple quantitative parameters, such as CT values on virtual non-contrast (VNC) images, iodine concentration (IC), electron density (ED), effective atomic number (Zeff), and CT values on virtual mono-energetic images (VMI), offering new insights for the diagnosis and evaluation of GISTs. ED is a crucial parameter in spectral CT, demonstrating unique advantages in reflecting tissue chemical composition and microstructure. Previous studies have demonstrated the clinical value of spectral CT quantitative parameters in predicting the differentiation of gastric cancer and rectal cancer deposits [10–12]. However, no comprehensive studies have investigated the use of ED for predicting risk stratification in GISTs, nor have they systematically evaluated the combined predictive value of spectral CT parameters and traditional CT features.

This study aims to develop and validate a multimodal predictive model incorporating dual-phase spectral CT parameters with key clinical-radiological features for preoperative risk stratification.

## Methods

### Patients

**Fig. 1:**
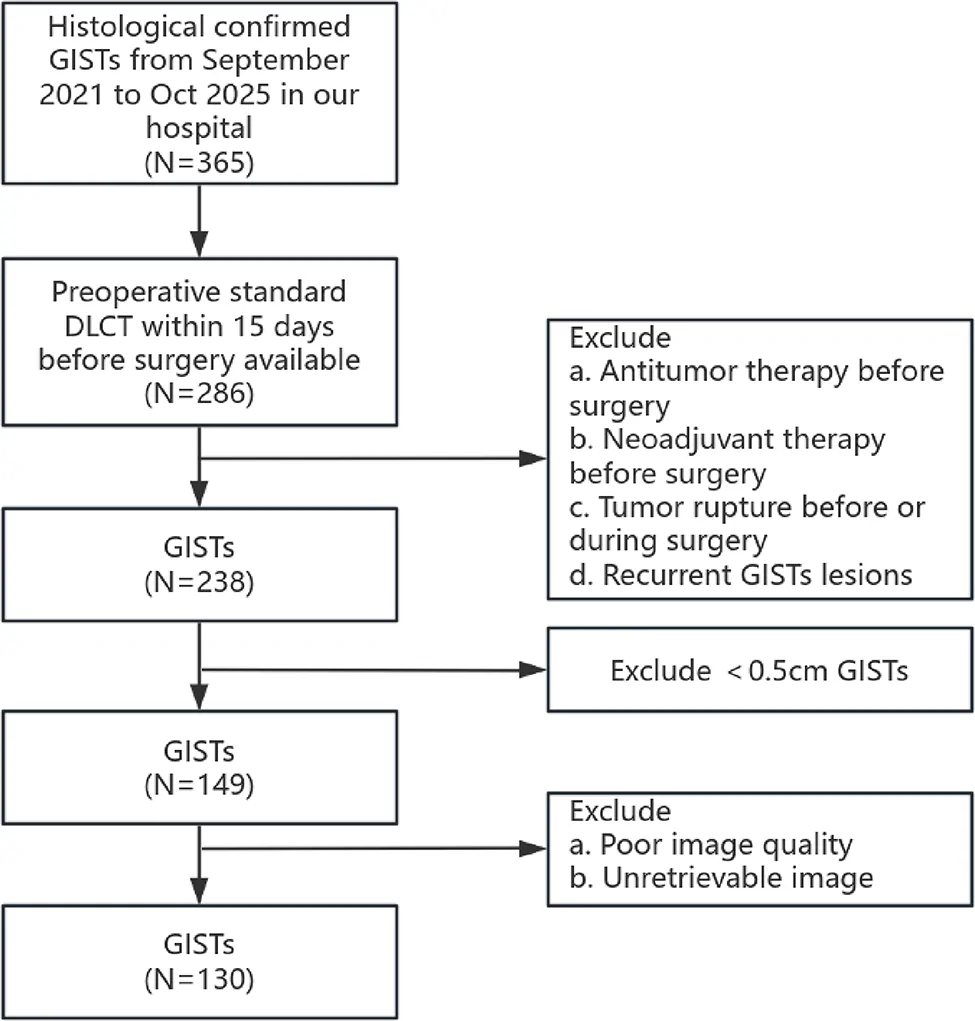
Flowchart of the patient selection. GISTs, Gastrointestinal Stromal Tumors; DLCT, Dual-layer spectral detector computed tomography

With the approval of the institutional review board and a waiver of informed consent, the retrospective study enrolled 365 patients with histologically confirmed gastrointestinal GISTs from September 2021 to October 2025 in our hospital. Final 130 patients (55 men and 75 women; mean age, 58.3 ± 11.6 years) met the inclusion criteria and were ultimately included in the analysis. The inclusion criteria were as follows: [1] Surgical pathology and immunohistochemistry confirmed GISTs with accurate risk stratification; [2] A standard contrast-enhanced DLCT examination was performed within 15 days before surgery; [3] No prior antitumor therapy before surgery. Exclusion criteria: [1] Patients received neoadjuvant therapy before surgery; [2] Patients had tumor rupture before or during surgery; [3] Recurrent GIST lesions; [4] Poor image quality unsuitable for analysis; [5] Maximum tumor diameter < 0.5 cm. The detailed research flowchart is shown in Fig. 1.

The demographic and clinicopathological data, including age, sex, tumor location, tumor size, mitotic counts, and distant metastasis were derived from medical records. The modified NIH criteria were applied to stratify the malignant potential based on tumor size and mitotic count. We further categorized very low- and low-risk patients were classified into the low-grade group, and intermediate- and high-risk patients were classified into the high-grade group. The 130 patients were subsequently grouped into the low- and high-grade malignant potential groups with a distribution of 83/47 (low/high).

### Spectral CT protocols

Patients were instructed to fast for at least 6 h before the examination. To achieve gastric distension, patients were asked to drink 500–800 mL of warm water immediately before scanning. No routine antiperistaltic (hypotonic) agent was administered. Abdominal contrast-enhanced scans were performed using a dual-layer detector spectral CT scanner (IQon, Philips Healthcare). The scanning range extended from the diaphragmatic dome to the level of the bilateral ischial tuberosities. Scanning parameters: Tube voltage 120 keV, automatic tube current modulation, matrix 512 × 512, pitch 1.016, slice thickness and spacing 1 mm. Iodinated contrast agent (iodine concentration 300 mg I/mL) was injected using a dual-syringe high-pressure injector at a flow rate of 3.0 mL/s and a dose of 1.5 mL/kg, followed by a 20-mL saline flush at the same flow rate. After contrast injection, arterial phase scans were performed at 30 s and venous phase scans at 60 s, respectively. A true pre-contrast (non-contrast) acquisition was performed before contrast administration, and virtual non-contrast (VNC) images were additionally reconstructed from the spectral acquisition. There is no statistically significant difference observed in CT attenuation values between VNC and true non-contrast images (*P* > 0.05), suggesting that VNC may serve as a potential surrogate for conventional non-contrast imaging.

### Imaging analysis

**Fig. 2:**
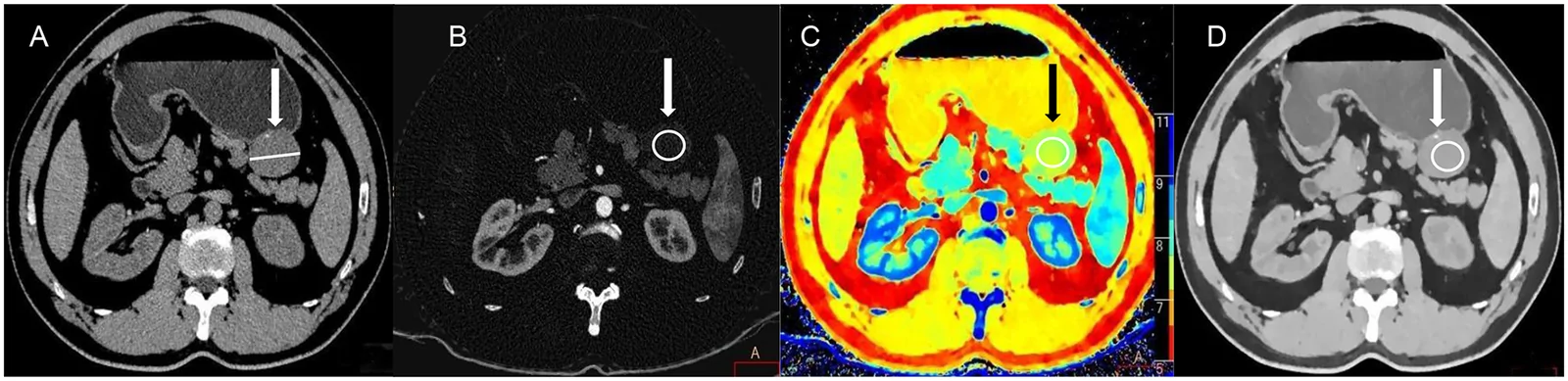
Example of ROI delineation in a patient with a pathologically confirmed low-risk gastric GIST (arrow), with a maximum tumor diameter of approximately 3.7 cm. **A**: Virtual on-contrast (VNC) value in arterial phase is 37.7; **B**: Iodine density map in arterial phase, lesion IC value 0.75 mg/ml, same-level abdominal aorta IC value 10.07 mg/ml, NIC 0.07 mg/ml; **C**: Effective atomic number (Zeff) map in arterial phase, Zeff 7.73; **D**: Electron density (ED) map in arterial phase, ED value approximately 103.9% EDW

After scanning, spectral-based images (SBIs) was reconstructed on Philips’s post-processing workstation (IntelliSpace Portal 10.1, Philips Healthcare). Virtual non-contrast (VNC) images, virtual mono-energetic images (VMIs) images, iodine concentration (IC) maps, effective atomic number (Zeff) maps, and electron density (ED) maps in arterial phase (AP) and venous phase (VP) were reconstructed. Regions of interest (ROIs) were manually drawn on the largest cross-section of the tumor, avoiding cystic or necrotic areas, and on the same level of the abdominal aorta or iliac artery. The ROI diameter for the tumor was greater than half of the minimum tumor diameter on the corresponding slice. All ROIs were measured twice, and the average values were recorded. Parameters included VNC, IC of the lesion and same-level aorta/iliac artery, ED, Zeff, and spectral curve slope (λ) (Fig. 2). Normalized iodine concentration (NIC) was calculated, NIC=$\:\frac{{IC}_{tumor}}{{IC}_{artery}}$, spectral curve slope (λ), λ=$\:\frac{{VMI}_{40keV}-{VMI}_{80keV}}{40}$, and dual-energy index (DEI) were derived, DEI=$\:\frac{{VMI}_{40keV}-{VMI}_{80keV}}{{VMI}_{40keV}+{VMI}_{80keV}+2000}$.

Two experienced abdominal imaging diagnosticians (with 5 and 10 years of abdominal imaging diagnostic experience, respectively) evaluated the morphological and quantitative data of the enrolled lesions using a double-blind method. In cases of disagreement, a consensus was reached through discussion. The following CT features were recorded: size (cm), location (gastric or non-gastric), EVFDM, necrosis, ulceration, calcification. Tumor size was defined as the maximal diameter on the transverse, coronal, or sagittal plane. EVFDM was defined as the presence of enlarged central or peripheral vessels feeding or draining the tumors [13]. Tumor necrosis was classified as absent/mild (< 20%) or prominent (> 20%) in the PVP [14]. Ulceration was considered present when a focal mucosal defect/indentation filled with air or fluid or when contrast material was found on the endoluminal surface of the lesion [15]. To assess interobserver reproducibility, both readers independently measured all spectral CT quantitative parameters (including ED, VNC, IC, Zeff, and λ) in a randomly selected subset of 30 patients. Interobserver agreement was evaluated using the intraclass correlation coefficient (ICC) with a two-way random-effects model. ICC values were interpreted as follows: <0.40, poor; 0.40–0.59, fair; 0.60–0.74, good; and 0.75–1.00, excellent agreement.

### Statistical analysis

Statistical analysis was performed using SPSS 23 and R software (version 4.2.1). Quantitative data were tested for normality using the Kolmogorov-Smirnov test. Normally distributed data were expressed as mean ± standard deviation, and intergroup comparisons were conducted using independent samples t-tests. Non-normally distributed data were expressed as median (Q1, Q3), and intergroup comparisons were performed using the Mann-Whitney U test. Least absolute shrinkage and selection operator (Lasso) was used to screen predictive indicators to build a logistic model. The predictive performance of the model was evaluated using ROC curves, calculating the area under the curve (AUC), sensitivity, specificity, accuracy. Decision curve analysis (DCA) was used to assess the net clinical benefits. And model’s calibration was visualized with a calibration curve plotted with 1000 times bootstrap resampling. P-value < 0.05 was considered statistically significant.

## Results

### Demographic, clinical, and pathologic characteristics of the patients

The clinical characteristics of the patients with GISTs are presented in Table 1. A total of 130 patients, aged 35–84 years (mean: 58.3 ± 11.6). The mean age was significantly lower in the low-risk group (*N* = 83, 56.6 ± 10.8 years) than in the high-risk group (*N* = 47, 61.3 ± 12.5 years) (t = 2.23, *P* = 0.03). Tumors were located in the stomach in 93 cases (71.5%) and non-gastric regions (small intestine: 22, colorectal: 13, esophagus: 2) in 37 cases (28.5%). In the low-risk group, 74 lesions (89.2%) were located in the stomach and 9 (10.8%) in non-gastric regions, whereas in the high-risk group, 19 lesions (40.4%) were gastric and 28 (59.6%) were non-gastric. This distribution showed a statistically significant difference ($\:\chi\:$^2^ = 35.0, *P* < 0.01). No significant difference in sex distribution was observed between the two groups (*P* > 0.05).

**Table 1:** Clinical and traditional CT characteristics of the GISTs patients

Characteristics	All (*N* = 130)	Low-risk(*N* = 83)	High-risk(*N* = 47)	*P* value
Age (mean ± SD)	58.3 ± 11.6	56.6 ± 10.8	61.3 ± 12.5	0.03*
Sex, no. (%)				0.43
Female	75(57.7%)	50(60.2%)	25(53.2%)	
Male	55(42.3%)	33(39.8%)	22(46.8%)	
Location, no. (%)				0.00*
Stomach	93(71.5%)	74(89.2%)	19(40.4%)	
Non-gastric regions	37(28.5%)	9(10.8%)	28(59.6%)	
Size (cm) (IQR)	3.0(1.4,6.0)	1.6(1.1,3.1)	6.7(4.2,8.6)	0.00*
Necrosis, no. (%)	44(33.8%)	10(12.0%)	34(72.3%)	0.00*
Ulceration, no. (%)	22(16.9%)	10(12.0%)	12(22.5%)	0.05
EVFDM, no. (%)	39(30.0%)	9(10.8%)	30(63.8%)	0.00*
Calcification, no. (%)	17(13.1%)	12(14.5%)	5(10.6%)	0.53

EVFDM, enlarged vessels feeding or draining the mass

* *p* < 0.05

### Association of CT features with the malignant potential of GISTs

In the low-risk group, 10 cases (10/83, 12.0%) exhibited necrosis, 12 (12/83, 14.5%) had calcification, 10 (10/83, 12.0%) showed ulceration, and 9 (9/83, 10.8%) displayed EVFDM. In the high-risk group, 34 cases (34/47, 72.3%) had necrosis, 5(5/47, 10.6%) had calcification, 12 (12/47, 22.5%) showed ulceration, and 30 (30/47, 63.8%) displayed EVFDM. The maximum tumor diameter ranged from 0.5 to 14 cm (mean: 3.0 cm [IQR, 1.4–6.0]). The median maximum tumor diameters in the low- risk groups were significantly smaller than that in high-risk groups (1.6 cm [IQR, 1.1–3.1] vs. 6.7 cm [IQR, 4.2–8.6]; *P* < 0.05). Necrosis occurrence rates were 12.0% and 72.3%, and EVFDM presence rates were 10.8% and 63.8% in the low- and high-risk groups respectively, all showing statistically significant differences (*P* < 0.05) (Table 1).

### Spectral CT quantitative parameter characteristics

The interobserver reproducibility for all spectral CT quantitative parameters was excellent, with ICCs ranging from 0.84 to 0.93. Specifically, the ICC for AP-ED was 0.90 (95% CI: 0.86–0.95), indicating excellent agreement between the two readers.

Significant differences were observed between the low- and high-risk groups in arterial-phase ED (104.3 ± 1.0 vs. 103.5 ± 0.5), VNC (39.7 ± 8.4 vs. 36.3 ± 7.1) (*P* < 0.05). No significant differences were found in other quantitative parameters (*P* > 0.05) (Table 2). To address the potential confounding effect of tumor location, a subgroup analysis stratified by tumor location (gastric vs. non-gastric) was performed. Among gastric GISTs (*n* = 93), AP-ED remained significantly different between low- and high-risk groups (104.0 ± 0.7 vs. 103.7 ± 0.5%EDW, *P* < 0.05). Similarly, among non-gastric GISTs (*n* = 37), the difference in AP-ED between low- and high-risk groups also showed statistical significance (104.8 ± 0.8 vs. 103.6 ± 0.5%EDW, *P* < 0.05). These results suggest that the observed ED difference is associated with malignant potential rather than being solely attributable to tumor location. Additionally, conventional arterial-phase CT attenuation values (HU) were compared between the two groups. The mean arterial-phase CT attenuation was 40.1 ± 3.2 HU in the low-risk group and 36.1 ± 5.6 HU in the high-risk group (*P* < 0.05). This finding suggests that the observed ED difference may partly reflect differences in contrast enhancement patterns between low- and high-risk GISTs, although ED as a spectral parameter provides complementary information beyond conventional HU values.

**Table 2:** Comparison of spectral CT quantitative parameters between low-risk and high-risk groups

Quantitative Parameter	Low-risk (*N* = 83)	High-risk (*N* = 47)	*P* value
AP-NIC (mg/ml)	0.07(0.03,0.11)	0.07(0.05,0.14)	0.25
AP-Zeff	7.84 ± 0.48	7.91 ± 0.50	0.57
AP-ED (%EDW)	104.3 ± 0.98	103.5 ± 0.53	0.00*
VP-NIC (mg/ml)	0.28(0.20,0.41)	0.33(0.20,0.43)	0.36
VP-Zeff	8.03 ± 0.39	8.12 ± 0.43	0.28
VP-ED (%EDW)	104.1 ± 1.02	103.9 ± 0.60	0.06
AP-VNC (HU)	39.7 ± 8.4	36.3 ± 7.1	0.03*
VP-VNC (HU)	38.5 ± 9.2	35.8 ± 6.4	0.05
AP- ${\lambda _{40 - 80Kev}}$	1.28(0.63,1.97)	1.21(0.79,3.14)	0.33
VP- ${\lambda _{40 - 80Kev}}$	2.14(1.29,2.95)	2.16(1.50,3.34)	0.31
AP- ${\rm{DE}}{{\rm{I}}_{40 - 80Kev}}$	0.03(0.01,0.04)	0.02(0.01,0.05)	0.32
VP- ${\rm{DE}}{{\rm{I}}_{40 - 80Kev}}$	0.04(0.02,0.05)	0.04(0.03,0.06)	0.29

NIC, Normalized iodine concentration; Zeff, Effective atomic number; ED, Electron density; VNC, CT values on virtual non-contrast images; DEI, Dual-energy index; AP, Arterial phase; VP, Venous phase

* Indicated *p* < 0.05

### Lasso variable screening model construction

**Fig. 3:**
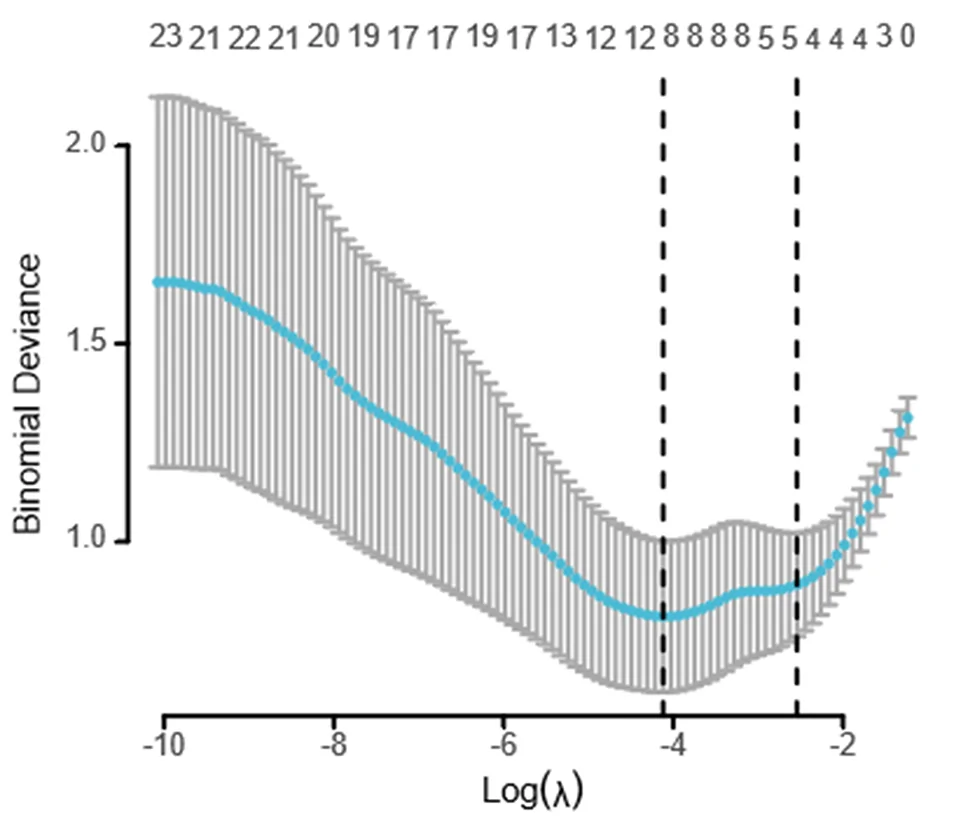
Coefficient screening plot of the Lasso regression model incorporating spectral CT quantitative parameters and clinical imaging features. The x-axis (bottom) represents log(λ), while the top x-axis indicates the number of non-zero coefficient variables for each lambda value. The left dashed line denotes the optimal lambda (lambda.min), with five variables selected

**Fig. 4:**
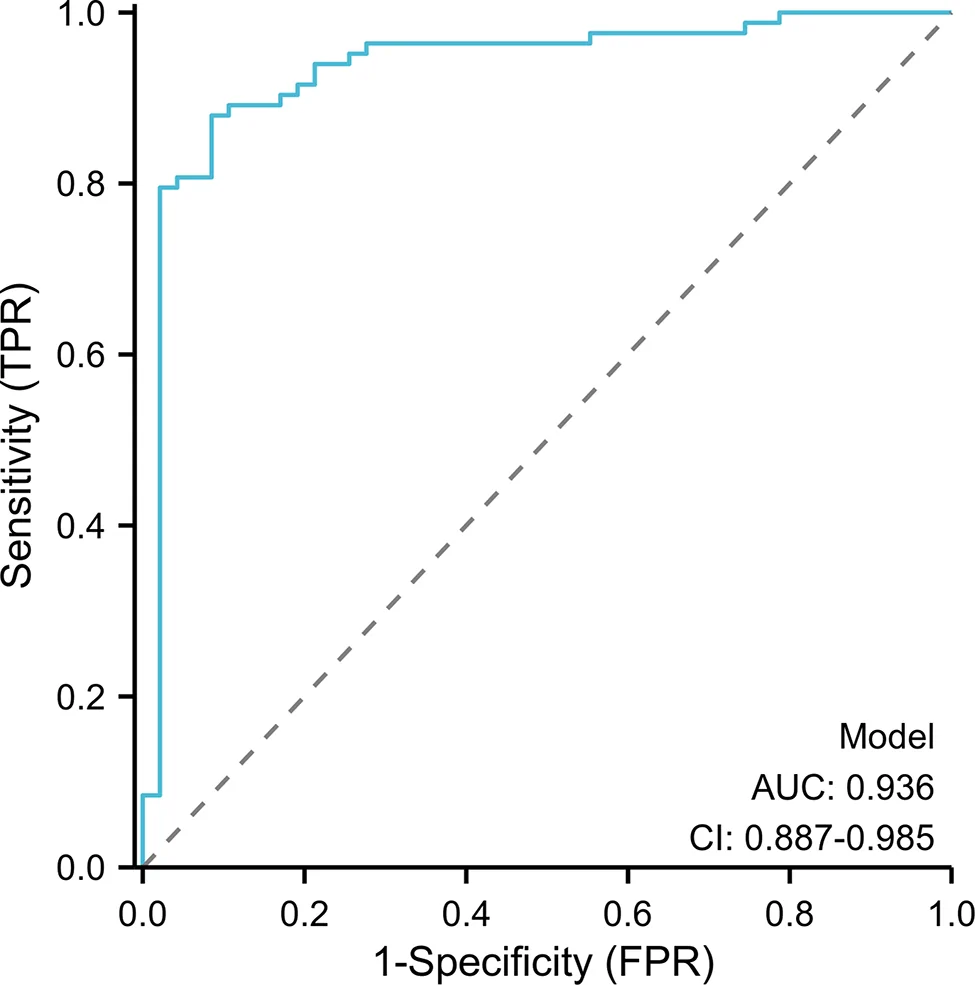
ROC curve of the Lasso regression model

**Fig. 5:**
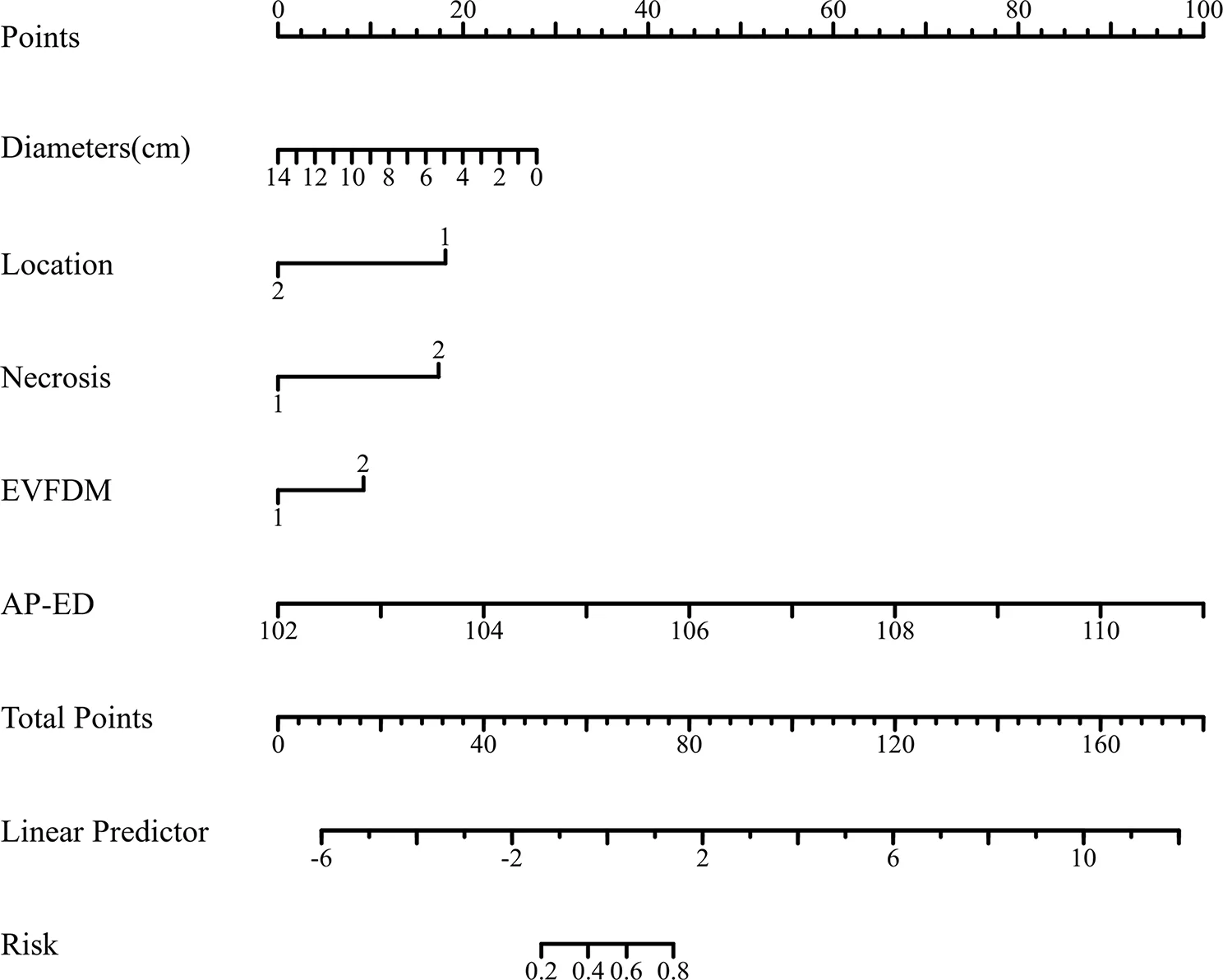
Nomogram for predicting GIST risk stratification using arterial-phase ED combined with maximum tumor diameter, lesion location, necrosis, and EVFDM

**Fig. 6:**
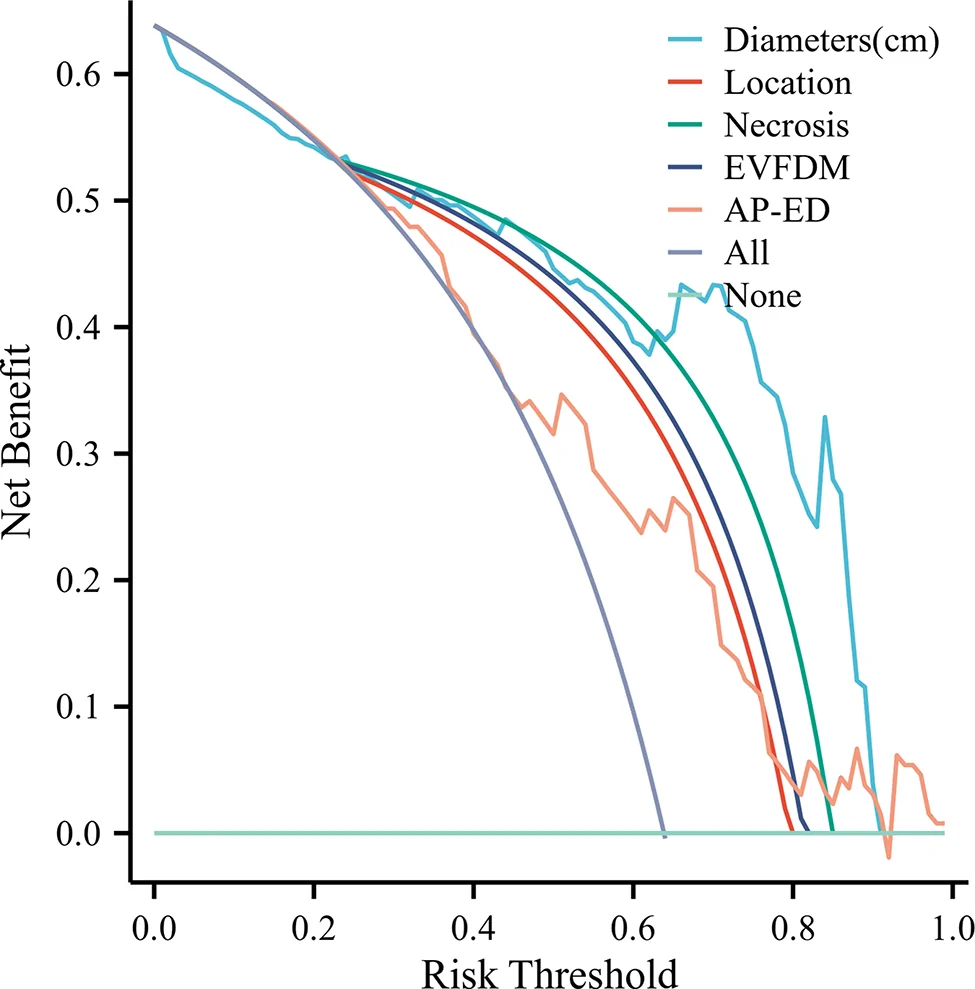
Graph shows the decision curve analysis (DCA) of the Lasso regression model

**Fig. 7:**
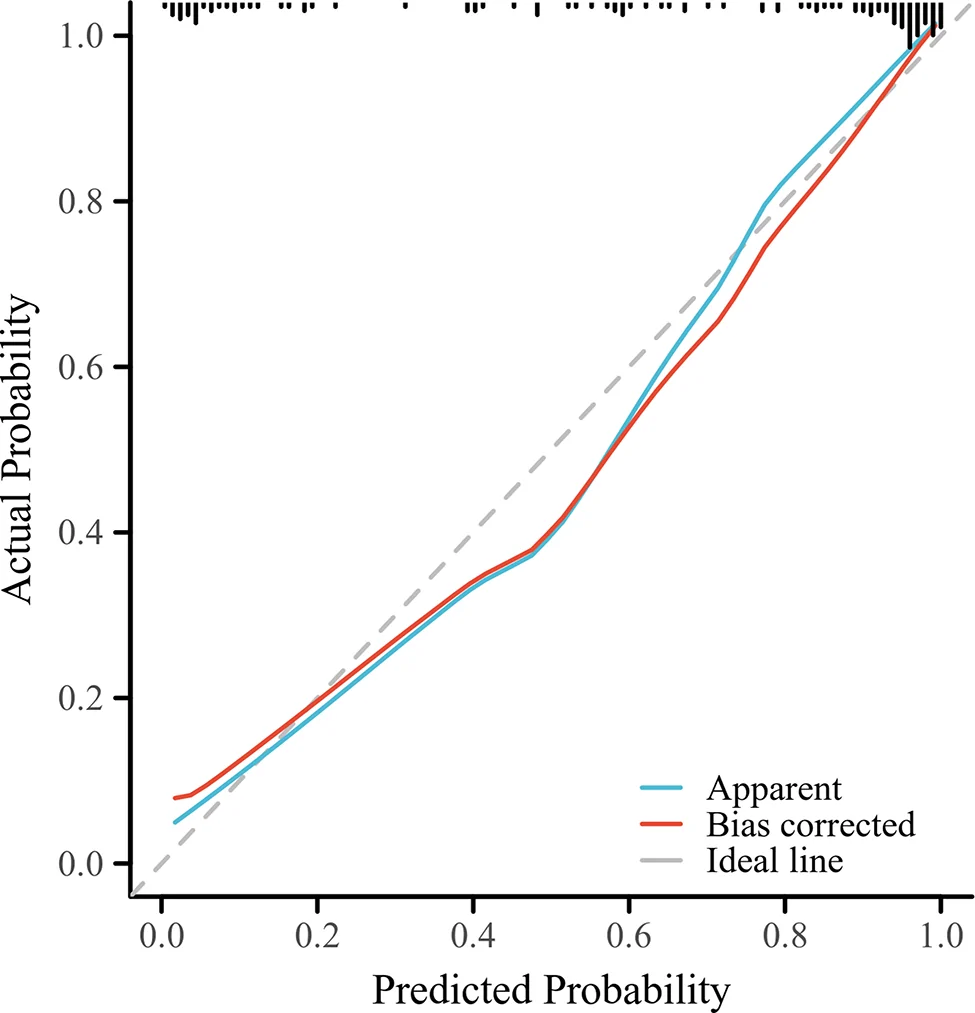
Graph shows the calibration curve of the Lasso regression model

All variables of clinical baseline characteristics, tumor morphology, and spectral CT parameters were further subjected to least absolute shrinkage and selection operator (LASSO) regression. The variables retained by the model included maximum tumor diameter, lesion location, necrosis, EVFDM, and arterial-phase ED (Fig. 3). The integrated model demonstrated strong performance with an AUC of 0.936 (95% CI: 0.887–0.985), sensitivity of 88.0%, specificity of 91.5%, and accuracy of 89.2% (Fig. 4), all of which outperformed those of the individual predictors (Table 3). The regression coefficients and odds ratios (ORs) with 95% CI for the final integrated model were presented in Table 4. A nomogram was constructed based on the Lasso model results (Fig. 5). Decision curve analysis (DCA) indicated that the integrated model provided a greater net clinical benefit than any single predictor (Fig. 6). The calibration plot of the integrated model revealed good predictive accuracy between the actual and predicted probabilities (Fig. 7). These results indicate that all five variables independently contributed to the prediction of high-risk GISTs. To evaluate the incremental value of spectral CT parameters, a model based solely on conventional CT features (tumor size, location, necrosis, and EVFDM) was compared with the integrated model including AP-ED. The conventional CT-only model yielded an AUC of 0.920 (95% CI: 0.870–0.970), while the integrated model achieved an AUC of 0.936 (95% CI: 0.887–0.985).

**Table 3:** ROC curve analysis and diagnostic performance of each variable in the Lasso Model

Variable	AUC	Sensitivity	Specificity
Maximum tumor diameter	0.886(0.826–0.947)	0.87	0.89
Lesion location	0.744(0.665–0.822)	0.89	0.59
Necrosis	0.801(0.728–0.875)	0.88	0.72
EVFDM	0.765(0.688–0.842)	0.89	0.64
AP-ED	0.736(0.648–0.824)	0.64	0.78
Integrated model	0.936(0.887–0.985)	0.88	0.92

**Table 4:** The regression coefficients and odds ratios (ORs) with 95% CI for the final integrated model

Characteristics	Odds Ratio (95% CI)	*P* value
Diameters(cm)	0.806 (0.663–0.980)	0.031
Location	0.142 (0.036–0.553)	0.005
Necrosis	6.505 (2.064–20.501)	0.001
EVFDM	2.710 (1.155–6.360)	0.022
AP-ED	3.318 (1.168–9.426)	0.024

## Discussion

This study established that a combined model integrating quantitative ED with tumor morphological features (tumor diameter, location, necrosis, and EVFDM) achieves superior performance (AUC 0.936), providing a novel non-invasive method for preoperative risk stratification. Two critical DLCT discoveries were validated: High-risk GISTs exhibited significantly lower arterial-phase ED than low-risk GISTs (103.5 ± 0.5 vs. 104.3 ± 1.0%EDW, *p* < 0.05), with 103.9%EDW identified as the optimal diagnostic threshold. Arterial-phase ED outperforms venous-phase parameters, indicating phase-specific biological significance. However, it should be acknowledged that the absolute difference in arterial-phase ED between the low- and high-risk groups was relatively small (< 1%EDW). While this difference reached statistical significance, its clinical applicability and the robustness of the proposed cutoff value (103.9%EDW) require further validation in larger, multicenter cohorts. ED should be interpreted as a complementary parameter within the integrated model rather than as a standalone diagnostic criterion.

Early and accurate risk assessment of GISTs plays a crucial role in guiding optimal treatment selection and the timing of targeted drug application. Clinical practice tends to favor tumor size for treatment decisions, with literature [16] suggesting that tumor diameter as an independent risk factor. However, previous studies [17] show that small tumors can exhibit pathological features similar to larger ones. Therefore, research on GISTs risk stratification remains highly significant, which primarily relies on pathological examination [18]. However, due to the limitations of biopsy sampling and intratumoral heterogeneity, identifying clinical and imaging biomarkers for preoperative risk stratification is particularly important.

Contrast-enhanced CT remains the preferred imaging modality for GISTs diagnosis and postoperative follow-up. Studies worldwide [19–21] confirm its value in GISTs risk stratification. However, the predictive model of our study had strong predictive efficacy, with an AUC of 0.936 (95% CI 0.887–0.985). This outperformed the AUC of models based on conventional contrast-enhanced CT combined with texture analysis (0.878, 95% CI 0.797–0.959) [22] and clinical models derived from traditional CT radiomics in training and testing cohorts (AUCs of 0.871 [95% CI: 0.811–0.931] and 0.854 [95% CI: 0.760–0.947], respectively) [23]. Larger tumor diameter, necrotic features, EVFDM, and non-gastric location were all associated with higher GISTs risk, consistent with previous studies [24, 25]. While some studies [19, 26] identified ulceration as an independent predictor of high-risk GISTs, our study found no statistical difference in ulceration between high- and low-risk groups, possibly due to the low incidence of ulceration in our cohort (16.9%, 22/130). It should be noted that tumor size, as one of the strongest predictors in our model, is also a major component of the modified NIH risk classification used as the reference standard. Consequently, a substantial proportion of the model’s predictive performance is inherently attributable to this variable. However, the inclusion of spectral CT-derived ED and other morphological features (necrosis, EVFDM, and location) provides additional predictive value beyond tumor size alone, as demonstrated by the incremental analysis comparing the conventional CT-only model with the integrated model.

DLCT electron density mapping visualizes the relative distribution of electron density at each voxel, revealing material properties difficult to assess with conventional CT. Currently, few studies have explored ED combined with clinical-imaging features for GISTs risk stratification. Our study is the first to establish the potential value of ED in GISTs risk assessment. We found that high-risk GISTs exhibited lower arterial-phase ED values than low-risk GISTs, with an optimal cutoff of 103.9 (%EDW) in the predictive model, achieving sensitivity, specificity, and accuracy of 88.0%, 91.5%, and 89.2% respectively. The inverse correlation between ED and malignant potential may reflect two pathophysiological processes: ⑴Angiogenic dysregulation: High-risk GISTs exhibit chaotic neovascularization (supported by higher EVFDM prevalence), diluting contrast media distribution and reducing ED values. ⑵ Cellular dehydration: Malignant transformation increases sodium-potassium pump activity, decreasing intracellular water content. Previous study on pancreatic ductal adenocarcinoma [27] found that ED was an independent risk factor for early postoperative recurrence and negatively correlated with it. These findings suggest that ED may reflect the biological behavior and malignancy of different tumor types, potentially serving as a potential imaging biomarker for GISTs risk stratification. Furthermore, since ED was measured during the arterial phase, it may be influenced by contrast enhancement and patient hemodynamics. The reported conventional CT attenuation values (HU) in both groups provide additional context for interpreting the ED differences, helping to distinguish whether the observed ED difference reflects enhancement-related effects or intrinsic tissue characteristics.

From a clinical perspective, the preoperative identification of high-risk GISTs using this integrated model may have several important implications. First, for patients predicted as high-risk, neoadjuvant imatinib therapy could be considered, as it has been shown to reduce tumor volume, facilitate complete surgical resection, and decrease the risk of intraoperative tumor rupture. Second, knowledge of the preoperative risk status may influence surgical planning, including the extent of gastric resection and the choice between endoscopic, laparoscopic, or open surgical approaches. Preoperative risk stratification could assist in selecting the most appropriate surgical strategy and surgical approach, while the primary surgical objective remains complete (R0) resection. Third, the model may aid in determining the appropriate postoperative surveillance strategy, with high-risk patients requiring more frequent follow-up imaging. Additionally, preoperative risk stratification may support referral decisions to specialized centers for complex cases. However, these potential clinical applications require prospective validation before implementation in routine clinical practice.

Although this study demonstrates encouraging performance, several limitations should be acknowledged. First, this study is a single-center retrospective analysis with a relatively limited sample size, which may affect the generalizability of the findings. Future research should involve multicenter, large-scale prospective validation to provide more robust clinical evidence. Second, ED measurements were obtained during the arterial phase (acquired at 30s post-injection), which may be influenced by variations in patients’ circulatory function. Third, quantitative measurements were obtained from ROIs drawn on the largest cross-sectional image of the tumor, which may not fully capture the heterogeneity of larger or necrotic lesions. Whole-tumor volumetric analysis in future studies may provide more comprehensive assessment of tumor biological characteristics. Fourth, this study was performed using a single dual-layer spectral CT platform (Philips IQon). Since quantitative spectral CT parameters may vary across different dual-energy CT technologies and vendors, the generalizability of these findings to other platforms remains to be determined. Fifth, although bootstrap calibration was performed, the predictive model was both developed and evaluated within the same patient cohort without an independent external validation cohort. Prospective multicenter validation is essential before routine clinical implementation.

## Conclusion

The integration of spectral CT-derived ED with conventional imaging criteria delivers a robust, non-invasive tool for preoperative risk stratification. These findings may assist clinicians in identifying high-risk individuals prior to treatment, thereby improving personalized decision-making.

## Data Availability

The datasets used and/or analyzed during the current study available from the corresponding author on reasonable request.

## Abbreviations

DLCT: Dual-layer spectral detector computed tomography
VNC: Virtual non-contrast
IC: Iodine concentration
ED: Electron density
Zeff: Effective atomic number
VMI: Virtual mono-energetic images
GISTs: Gastrointestinal stromal tumors
AP: Arterial phase
VP: Venous phase
Lasso: Least absolute shrinkage and selection operator
ROC: Receiver operating characteristic
DCA: Decision curve analysis
EVFDM: Enlarged feeding/draining vessels
AUC: Area under the curve
CI: Confidence Interval
